# Special Issue “Mechanisms of Neurotoxicity”

**DOI:** 10.3390/ijms26209992

**Published:** 2025-10-14

**Authors:** Paola Alberti, Eleonora Pozzi

**Affiliations:** 1 Experimental Neurology Unit, School of Medicine and Surgery, University of Milani-Bicocca, 20900 Monza, Italy; eleonora.pozzi@unimib.it; 2 NeuroMI (Milan Center for Neuroscience), 20126 Milan, Italy

An exciting and relevant topic is addressed in this paper collection encompassing both peripheral and central nervous system mechanisms of damage. This is of particular interest since neurons are a perennial cell population and, therefore, neurotoxicity understanding and management is a relevant challenge to treat/prevent neurological disorders [1,2,3]. There are many different potentially neurotoxic agents which include chemotherapy drugs, environmental pollution, et cetera. Mechanisms of damage that involve the peripheral and/or central nervous system are explored to pave the way to potential treatment strategies relying on a robust biological rational.

Since neurons are excitable cells, ion channels/transporters can be a pivotal element leading to axonal damage and neuronal death [4,5]. A clear-cut review of their involvement, exploiting as a playground chemotherapy-induced peripheral neurotoxicity (CIPN) is provided as well as an in depth reasoning on how ion channels/transporters are quire susceptible, in their functioning, to changes in the environment the cell is exposed to which can be also triggering neurotoxicity.

The central nervous system is not overlooked too in this paper collection, as mentioned above, and the role of excitotoxicity [6,7] is also dissected in the in depth in the neurodegenerative disorder and in pain modulation.

Another topic that is presented via research data is the role of oxidative stress in determining alterations of the nervous system [8,9,10,11], exploiting zebrafish models, as well as its role in determining neurotoxicity acting against glial cells.

Last, but not least, our Special Issue is enriched by a detailed review of mechanisms leading to an entity that is becoming more and more relevant: HIV-associated neurocognitive disorder (HAND) [12,13].
